# BRENDA in 2026: a Global Core Biodata Resource for functional enzyme and metabolic data within the DSMZ Digital Diversity

**DOI:** 10.1093/nar/gkaf1113

**Published:** 2025-11-06

**Authors:** Julia Hauenstein, Lisa Jeske, Antje Jäde, Mathias Krull, Katrin Dümmer, Julia Koblitz, Anja Tietz, Dieter Jahn, Lorenz Christian Reimer, Boyke Bunk

**Affiliations:** Leibniz Institute DSMZ-German Collection of Microorganisms and Cell Cultures, Inhoffenstraße 7 B, 38124 Braunschweig, Germany; Leibniz Institute DSMZ-German Collection of Microorganisms and Cell Cultures, Inhoffenstraße 7 B, 38124 Braunschweig, Germany; Leibniz Institute DSMZ-German Collection of Microorganisms and Cell Cultures, Inhoffenstraße 7 B, 38124 Braunschweig, Germany; Leibniz Institute DSMZ-German Collection of Microorganisms and Cell Cultures, Inhoffenstraße 7 B, 38124 Braunschweig, Germany; Leibniz Institute DSMZ-German Collection of Microorganisms and Cell Cultures, Inhoffenstraße 7 B, 38124 Braunschweig, Germany; Leibniz Institute DSMZ-German Collection of Microorganisms and Cell Cultures, Inhoffenstraße 7 B, 38124 Braunschweig, Germany; Leibniz Institute DSMZ-German Collection of Microorganisms and Cell Cultures, Inhoffenstraße 7 B, 38124 Braunschweig, Germany; Braunschweig Integrated Centre of Systems Biology (BRICS), Braunschweig University of Technology, Rebenring 56, 38106 Braunschweig, Germany; Leibniz Institute DSMZ-German Collection of Microorganisms and Cell Cultures, Inhoffenstraße 7 B, 38124 Braunschweig, Germany; Leibniz Institute DSMZ-German Collection of Microorganisms and Cell Cultures, Inhoffenstraße 7 B, 38124 Braunschweig, Germany

## Abstract

BRENDA (https://www.brenda-enzymes.org/), the most comprehensive enzyme and ligand database for over nearly four decades, has seen major developments since 2021, further solidifying its role as an *ELIXIR Core Data Resource* and *Global Core Biodata Resource* in the life sciences. As part of the DSMZ Digital Diversity consortium (https://hub.dsmz.de) since 2023, BRENDA has introduced a prototype knowledge graph accessible via a public SPARQL endpoint, enabling semantic search, data integration, and improved reusability in line with FAIR principles. The database now hosts ∼5.8 million data points on 112 288 enzymes from 15 335 organisms and 173 164 references, alongside 278 840 ligands. Recent updates include the manual annotation of ∼16 000 references, the addition of 615 EC numbers—480 with newly curated data—and revisions to 4422 EC classes. The curated pathway collection has expanded to 195 metabolic maps, now supported by interactive pathway summary pages that organize key information into structured, user-friendly views. BRENDA now also incorporates DSMZCellDive data, visually linking enzyme classes to high-expressing human cell lines. A new gene-centric search interface allows streamlined access to enzyme-specific data using gene symbols or identifiers. These enhancements advance BRENDA’s interoperability and analytical capabilities, reinforcing its integration with major bioinformatics resources such as SILVA, BacDive, and LPSN.

## Graphical Abstract

**Graphical Abstract:**
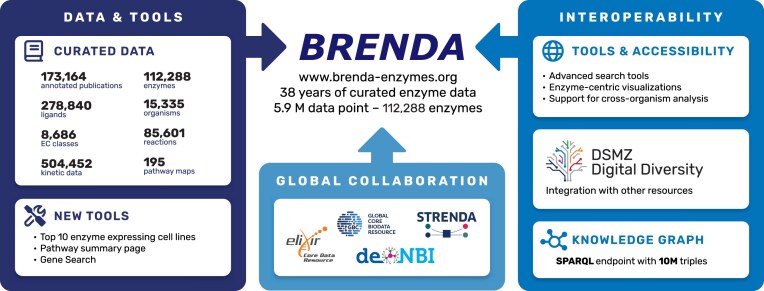

## Introduction

Enzymes are essential catalysts of life, playing pivotal roles in metabolic processes across all domains of biology. As the life sciences advance at an unprecedented pace—driven by high-throughput sequencing, proteomics, interactomics, metabolomics, integrative systems biology, and artificial intelligence—access to high-quality, curated biochemical data has never been more critical. One of the most important resources enabling these modelling processes allowing for a holistic cellular view is BRENDA (BRaunschweig ENzyme DAtabase, www.brenda-enzymes.org), the comprehensive information system on enzymes and enzyme–ligand interactions.

At the beginning of 2015, BRENDA was a key service within the German Network for Bioinformatics Infrastructure (de.NBI), supporting national and European life science research. In 2018, BRENDA was selected as an ELIXIR Core Data Resource [1, 2], acknowledging its fundamental importance to the European life science data infrastructure. Further reinforcing its global role, BRENDA was recognized in 2022 as a Global Core Biodata Resource [3], a designation reserved for data resources of critical importance to the broader biological and biomedical community worldwide.

Enzyme data in BRENDA are classified according to the Enzyme Commission (EC) nomenclature of the IUBMB (International Union of Biochemistry and Molecular Biology) [4]. The database currently contains over 5.8 million manually evaluated data points from 173 164 primary literature references, covering 112 288 enzymes from 15 335 organisms. In addition to manually curated data, BRENDA incorporates results from text and data mining, external sources, and prediction algorithms. These provide insights into disease associations, protein sequences, 3D structures, predicted enzyme localization, and genome annotations. A key component of BRENDA is its ligand database, which includes all compounds that interact with enzymes—such as substrates, products, inhibitors, cofactors, and activators. A total of 278 840 molecules includes small and macromolecules, each documented with names and synonyms to ensure consistency in referencing.

To facilitate user access and exploration, BRENDA supports a wide range of query options, including quick searches, full-text searches, advanced filters, and structure-based ligand queries using a built-in molecule editor for substructure searches. Visualization tools such as revised BRENDA Pathway Maps, 3D enzyme structure views, and word maps enhance user understanding by presenting complex data in intuitive formats. BRENDA includes and links to a wide range of external resources such as IUBMB ExplorEnz, PubMed [5], UniProt [6], PDB [7], BacDive [8], NCBI MeSH [5], PROSITE [9], and InterPro [10], further expanding the utility and reach of the database.

In addition to enzyme data, BRENDA offers genome, taxonomy, and ontology resources, including the BRENDA Tissue Ontology (BTO) [11], a comprehensive vocabulary of tissues and cell lines established in 2003. The BTO includes synonyms, definitions, and links to enzyme data for specific body parts, tissues, and cells, and is interoperable with major ontologies such as the Gene Ontology.

In 2023, BRENDA became an integral part of the newly founded DSMZ Digital Diversity framework. The Leibniz Institute DSMZ–German Collection of Microorganisms and Cell Cultures, a leading infrastructure for microbial biodiversity and data-driven microbiology, is building a semantically enriched, interconnected data consortium.

This publication presents an overview of BRENDA’s current scope, data architecture, and role within the DSMZ’s digital infrastructure. It highlights alignment with the FAIR data principles (findable, accessible, interoperable, and reusable) and emphasizes the importance of continued high-quality data integration and curation to support the needs of the scientific community.

Since the last publication in Nucleic Acids Research, seven major database updates (2021.1 to 2025.1) have been made available online. In addition, several new features have been implemented, including a new visualization on the Enzyme Summary Page and the “Highest Expressing Human Cell Lines” feature, which highlights cell lines with the highest enzyme expression. A new search option, the Gene Search, has been introduced to provide users with an additional entry point. Furthermore, the existing Enzyme, Ligand, Organism, and Literature Summary Pages have been extended with the addition of a Pathway Summary Page. Complementing these developments, BRENDA has also been expanded with a first version of a dedicated knowledge graph that integrates its content into the wider DSMZ Digital Diversity ecosystem.

## BRENDA in the DSMZ digital diversity consortium

Since 2023, BRENDA has been part of the DSMZ Digital Diversity consortium, which currently encompasses the development of nine life science databases, four of which are Global Core Biodata Resources, at the Leibniz Institute DSMZ–German Collection of Microorganisms and Cell Cultures in Germany. This relocation to the DSMZ, one of the world’s most extensive repositories of microorganisms and cell cultures, signifies a crucial step toward securing constant development and ensuring BRENDA’s continued role as a core data resource in the life sciences. The move allows strategic planning to modernize both the database and its services. Additionally, by integrating BRENDA into the DSMZ Digital Diversity Hub (D3; https://hub.dsmz.de), a platform that consolidates various scientific databases at DSMZ, we aim to enhance interoperability and foster connections with other DSMZ resources, such as BacDive, LPSN, TYGS [12], SILVA [13], CellDive [14], Media*Dive* [15], Phage*Dive* [16], and StrainInfo (https://straininfo.dsmz.de). The primary objective is to offer integrated access to data spanning a wide array of biological entities, thereby supporting innovative research and discoveries. The integration of cell line expression data from DSMZCellDive with BRENDA data enables the identification of the highest expressing human cell lines on the enzyme summary pages (see the “Highest expressing human cell lines” section). To facilitate efficient querying, integration, and interpretation of data across DSMZ Digital Diversity, a knowledge graph was established via a newly developed SPARQL endpoint (see the “BRENDA knowledge graph” section). As an additional feature, a pathway summary page serves as an endpoint for the BRENDA knowledge graph (see the “Pathway summary page” section). To further expand these existing integrations, we plan to connect enzymatic data from BRENDA to SILVA’s taxonomic and rRNA sequence data. This may help investigate metabolic patterns in different organisms and deepen our insight into the evolutionary relationships of enzymatic functions across various taxa. To allow users to easily navigate between the different data of the resources and to connect the data of the different resources, an integrated search has been developed across all DSMZ databases (https://hub.dsmz.de/#/search).

## BRENDA content—status quo and new data

The main quality feature of the BRENDA enzyme database is the manual curation of scientific publications. With the latest update release 2025.1, detailed information on 8698 enzyme classes (EC numbers) is provided. Among these, 8274 correspond to officially accepted EC numbers as approved by the IUBMB and sourced from their ExplorEnz database. In addition to the categorized EC numbers, BRENDA also provides “preliminary BRENDA-supplied EC numbers” that are still awaiting approval by the IUBMB. Since the last publication in 2021, 614 new EC classes have been added. Of these, 480 EC numbers contain newly curated manual annotations, 104 were created by transferring data from other or preliminary EC numbers, and for 31 EC numbers the newly curated data will be included in the upcoming BRENDA release 2026.1. The pathway overview map currently contains 195 metabolic pathways of different sizes, with 26 new ones since the last publication. The pathways comprise a total of 12 016 nodes representing 2110 different enzymes, 2338 different metabolites, and 2892 reactions.

The manual annotation procedure in BRENDA covers ~60 distinct data fields. These fields encompass a wide range of life science information, especially in the context of biochemistry, molecular biology, and biotechnology from all taxonomic groups. The data fields contain data of reactions and specificity, occurrence, protein and enzyme–ligand interaction, protein structure, stability and sensitivity, and kinetic data, including experimental conditions. Each data point, apart from the information provided directly by the IUBMB, is meticulously linked to the organism of origin, relevant literature references, and, where available, the UniProt protein sequence ID.

BRENDA undergoes continuous quality control to ensure the accuracy of its content. Typical corrections include the removal of redundant references, revisions of scientific organism names, adjustments of incorrect molecular structures, misassigned localizations, tissues, or enzymes, unbalanced reactions, erroneous enzyme names, and broken links, as well as updates to PubMed IDs, journal names, and UniProt accessions. These corrections are guided by automated check scripts that regularly flag potential errors, which are then reviewed and validated by expert curators against the primary literature before being updated in the database.

Release 2025.1 covers data extracted since 2023. Some releases were combined due to the migration process to the DSMZ, ensuring seamless transition and continuous data integration. Since the last publication in 2021, ∼16 000 new references have been manually annotated, and 4 422 EC classes were updated. Table 1 shows the development of data content for a selection of data fields from the last paper in 2021 (Release 2020.2) to the last release 2025.1.

**Table 1.** Number of entries in selected data fields

Data field	Release 2020.2	Release 2024.1	Release 2025.1	Increase since 2020.2
EC classes	8084	8476	8698	+614
Enzymes	97 116	106 313	112 288	+15 172
Kinetic data	438 027	480 240	504 452	+66 425
Ligands	240 031	262 180	278 840	+38 809
Organisms	13 196	14 290	15 335	+2139
Reactions	75 723	82 172	85 601	+9878
References	156 930	166 307	173 164	+16 234
Total data points	5 047 969	5 557 493	5 822 081	+774 112

The numbers refer to the combination of enzyme protein, source organism, and, in case of kinetic data, a literature reference. The term “enzymes” refers either to a protein sequence or to a protein isolated from a given organism without its sequence having been determined.

## New developments and major improvements

### BRENDA knowledge graph

Knowledge graphs have become an increasingly valuable resource within the landscape of biological data infrastructures. They enable efficient querying, integration, and interpretation of heterogeneous data sources by semantically structuring complex relationships between entities such as enzymes, compounds, pathways, and organisms. Unlike traditional databases, knowledge graphs offer inherent flexibility through their graph-based data model and ontology-driven semantics, allowing for dynamic expansion and interoperability. This flexibility is particularly beneficial in the life sciences, where data is diverse, constantly evolving, and often linked across multiple domains. Knowledge graphs facilitate advanced use cases such as hypothesis generation, semantic search, and automated reasoning. They also enhance findability and reusability, aligning with the FAIR data principles.

To build such graphs, data are commonly represented using the resource description framework (RDF). RDF stores information in simple sentences of the form subject–predicate–object (e.g. enzyme X–catalyze–reaction Y). By chaining many such triples together, RDF creates a graph that links biological entities and their relationships. Queries over these graphs are performed using SPARQL, the standard query language for RDF, which allows users to retrieve and combine the matching data from the graph [17].

SPARQL queries are primarily constructed by bioinformaticians and data scientists, who develop workflows and integrate BRENDA data with other resources, as well as by computational and systems biologists, who design queries to support pathway and network modeling. Other users such as biochemists, chemists, pharmacologists, biotechnologists, and medical researchers are expected to benefit in the future, once these query results are made accessible through user-friendly search and analysis tools. A major benefit for users arises from the interlinking of multiple knowledge graphs, such as those derived from BRENDA, BacDive, and UniProt, which enables the generation of novel insights beyond the scope of any individual resource.

**Figure 1.**
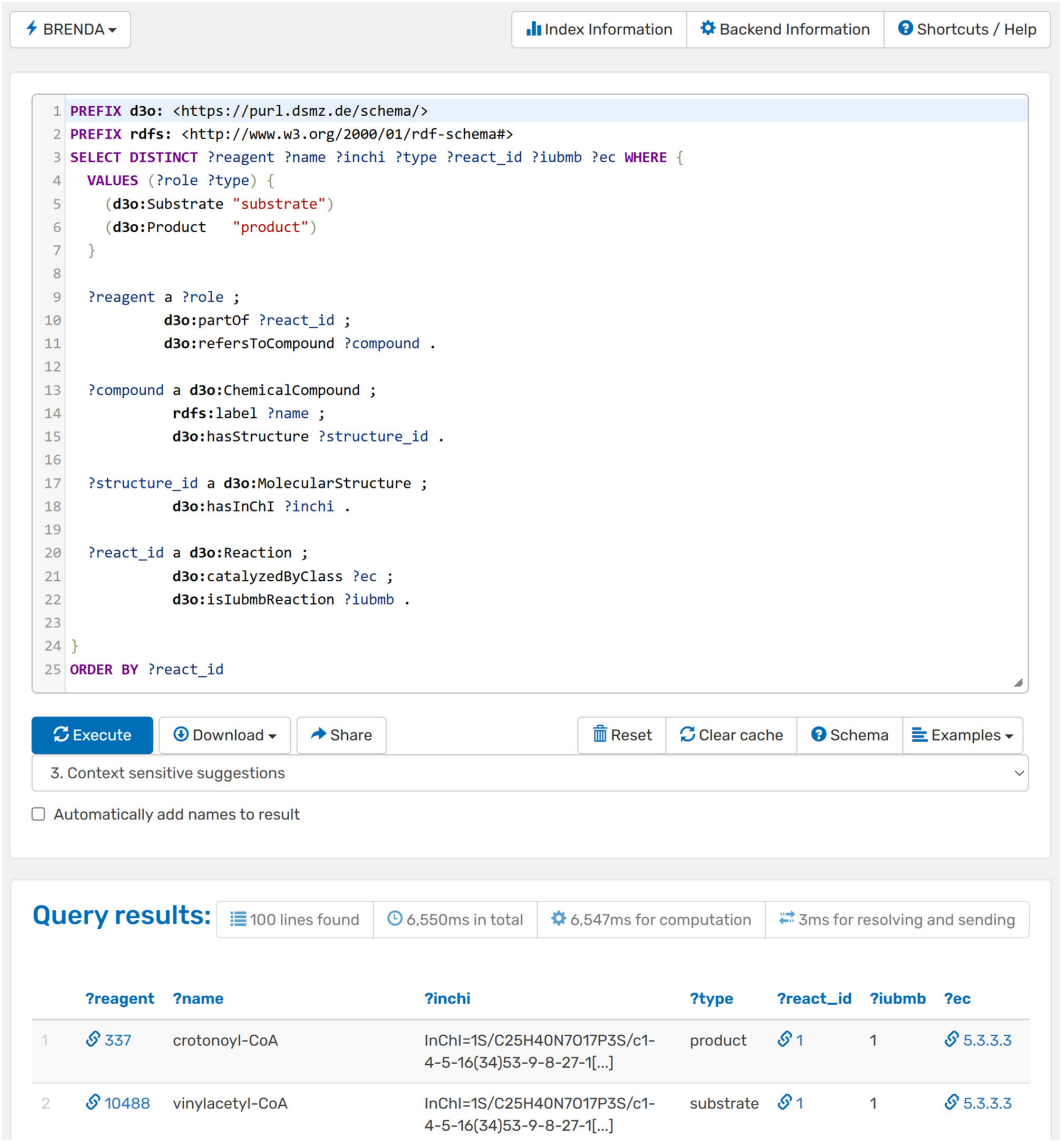
Example of a SPARQL query executed in the new BRENDA SPARQL interface. The query retrieves reaction data including substrates, products, compound identifiers (InChI), and associated EC numbers. The results section lists the retrieved compounds (e.g. crotonoyl-CoA, vinylacetyl-CoA) along with their role in the reaction.

A first prototype of a BRENDA knowledge graph has been published and is accessible via a newly established SPARQL endpoint at https://sparql.dsmz.de/brenda (Fig. 1). The knowledge graph interface can also be accessed directly from the BRENDA homepage. With the new SPARQL endpoint, a frequently requested use case has also been implemented. This use case was identified through a feedback and citation analysis, as described below. It comprises the mapping of EC numbers, reaction data, and structural information in the form of InChIs, and is illustrated in Fig. 1. The interface additionally provides example SPARQL queries under the Examples section, as well as an overview of the entities and their respective relations under schema. Built upon the DSMZ Digital Diversity Ontology (https://bioportal.bioontology.org/ontologies/D3O), the knowledge graph was implemented using R2RML [18] mappings, the Ontop framework [19], and the QLever engine [20]. The graph currently supports integration approaches within the previously described DSMZ Digital Diversity infrastructure. It contains structured information on entities such as *Activator, Cofactor, Compound, CompoundStructure, Enzyme* (linked to UniProt), *EnzymeClass, Inhibitor, Localization* (linked to the Gene Ontology [21]), *LocalizationOccurrence, Organism* (linked to the NCBI Taxonomy and Bac*Dive*), *Pathway, Reaction, Reference* (linked to PubMed and EuropePMC [22]), *Tissue* (linked to the BTO), and *TissueOccurrence. LocalizationOccurrence* and *TissueOccurrence* include links to *Reference, Enzyme*, and *Organism* entities. This initial release comprises a total of 10 798 737 RDF triples, and it will be continuously expanded until all qualified BRENDA data are represented within the knowledge graph. Future developments will include links to additional data sources such as LPSN, SILVA, and other databases hosted by the DSMZ. To further enhance the graph’s interoperability and value for cross-domain biological research, there will also be integration with other knowledge graphs, such as Ensembl [23] for gene-related data and ChEMBL [24] for compound information.

### Highest expressing human cell lines

**Figure 2.**
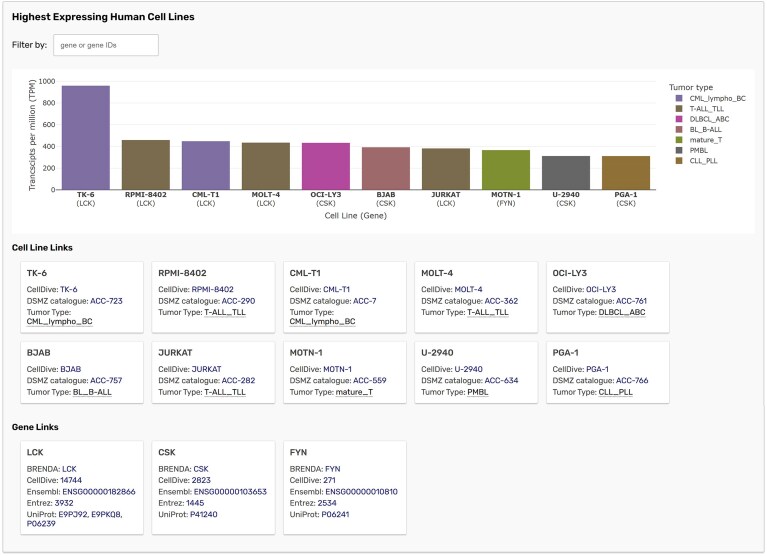
Highest expressing human cell lines for EC 2.7.10.2 with links to cell lines and genes (https://brenda-enzymes.org/enzyme.php?ecno=2.7.10.2#CELLLINES).

DSMZCellDive is a curated resource enabling researchers to select suitable model cell lines for their projects by determining RNA-Seq derived gene expression profiles and other properties from over 1 000 cell lines. To connect both databases, human cell lines with the highest expression values for human enzyme-encoding genes were integrated into BRENDA. The highest expressing cell lines are displayed on the enzyme summary pages in a bar chart for the genes belonging to the respective enzyme class (Fig. 2). The cell line bars are color-coded for belonging to certain tumor types and the diagram can be filtered by genes if the enzyme class contains more than one gene. Crosslinks to DSMZCellDive and comprehensive gene and protein data resources UniProtKB, NCBI Gene, and Ensembl are provided below the chart. As part of an *Enzyme Summary Page*, the highest expressing cell line data can be accessed primarily by searching for EC numbers, enzyme names, or by using the new *Gene Search* described below.

### Gene search

To allow a quick identification of the enzymatic properties of a gene, a new search option has been added to BRENDA (https://brenda-enzymes.org/genes.php). Available search fields include the short name (symbol), full name, organism, and a number of public database gene identifiers from e.g UniProtKB, NCBI Gene, and Ensembl. From the gene search results, the respective enzyme summary report as well as the expressing cell line section can be directly reached. Links to the main external gene data resources are also given. This search option is a first step towards establishing a stable gene endpoint in a future release of BRENDA.

### Pathway summary page

**Figure 3.**
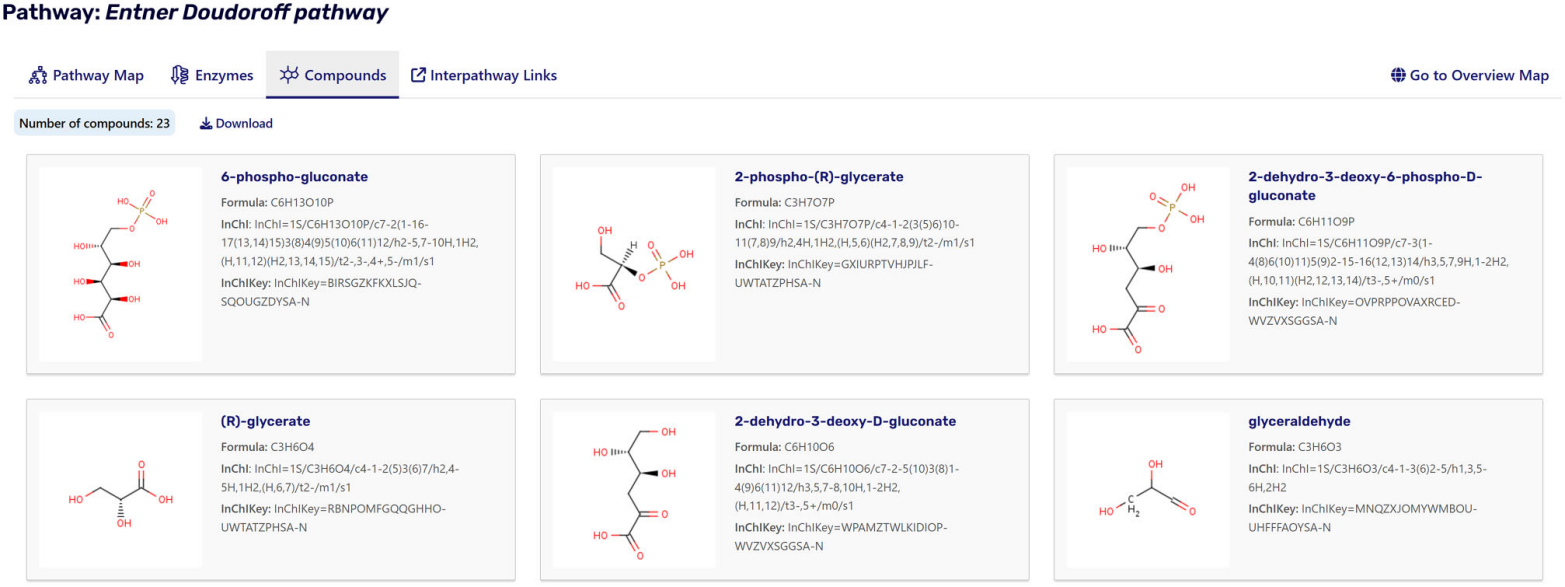
Compounds view of the pathway summary page of the Entner–Doudoroff pathway. The summary page provides access to different views, including the pathway map, enzymes, compounds, and interpathway links (https://brenda-enzymes.org/pathway.php?pathway=Entner Doudoroff pathway). Shown here is the Compounds section, which lists pathway metabolites along with their molecular structures, formulas, and identifiers (InChI and InChIKey).

Building upon the established metabolic pathways and to serve as a dedicated endpoint within the knowledge graph, the pathway summary page was developed. It provides a comprehensive and interactive overview of metabolic pathways, organizing key information into distinct tabs for clarity and ease of use. The overview tab presents a visual map of the pathway, while the enzymes tab lists all participating enzymes with a link to their enzyme summary pages. The metabolites tab details the compounds involved in the pathway (Fig. 3), including their formula, the InChl, the InChlKey, and a link to the corresponding ligand summary page. Additionally, the interpathway links tab highlights connections to related pathways, facilitating a broader understanding of metabolic networks and regulatory interactions. The data of each tab are downloadable for the user. By establishing a dedicated endpoint within BRENDA, pathway summary pages open the perspective for future interlinking with other databases and become powerful tools for comparative analysis, enabling researchers to efficiently navigate, visualize, and interrogate complex biological data. This structured approach supports the global scientific community in advancing discoveries in drug development, evolutionary biology, and metabolic engineering.

## Shaping the future: insights from usage, feedback, and citations

The development of BRENDA is guided by the needs and expectations of its user community. Analyses of usage behavior, citation patterns, and direct feedback provide insights into how the database is currently used and which features are considered important. These perspectives offer a valuable basis for targeted improvements.

### User statistics

**Figure 4.**
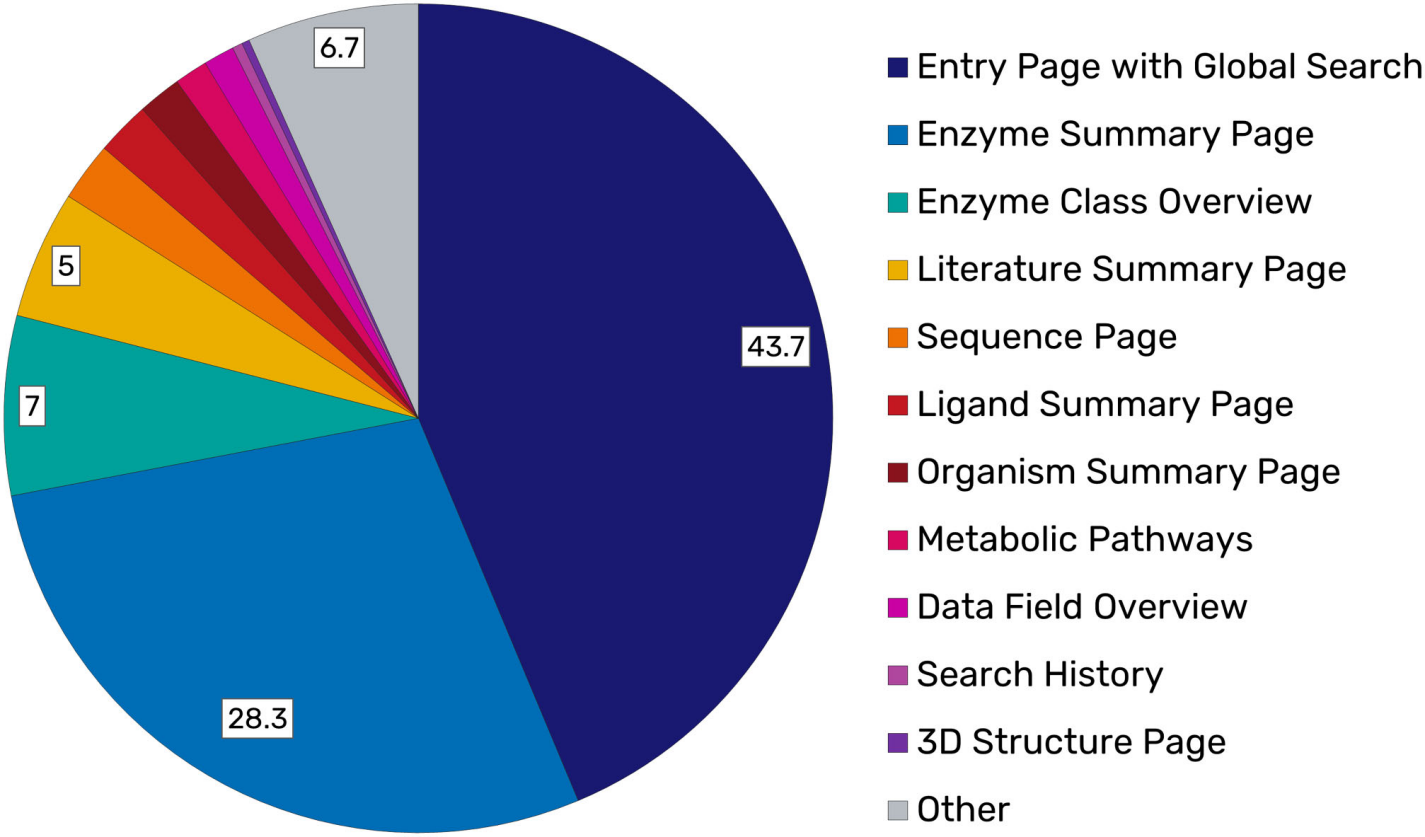
Distribution of page views in BRENDA showing the most frequently accessed sections.

User access patterns show a strong focus on a few core entry sites: the global search (43.7%) and the enzyme summary page (28.3%) dominate traffic, followed by moderate use of enzyme class overviews and literature summaries. Specialized sections such as sequence, ligand, and pathway pages attract considerably less attention, as illustrated in Fig. 4.

These insights highlight which content areas users rely on most and therefore should be prioritized in the upcoming redevelopment. To obtain these findings, unique pageviews across all sections of BRENDA were analyzed. Based on this, the new website will feature an improved global search, a consolidated enzyme class overview with IUBMB integration, and a revised data field overview. In addition, entirely new components are planned, including a help section and a statistics dashboard.

### Citation analysis

BRENDA is most frequently used for functional parameters such as kinetic values, metabolic reactions, ligands, and protein sequences. The database plays a central role in genome-scale metabolic modeling, flux balance analysis, machine learning approaches (including LLM training), and drug target identification.

These conclusions are derived from a citation analysis of 3498 publications referencing BRENDA, of which 382 were studied in detail. The analysis included keyword and context frequency counts as well as categorization of use cases. The results underline BRENDA’s role as a widely adopted and influential data source across diverse research domains.

### Feedback analysis

Direct input from users emphasizes three key requirements for future development: (i) seamless cheminformatics integration through SMILES notations, (ii) a modern and well-documented API, and (iii) enhanced usability of the search interface. Users also requested more flexible data download options and improved data quality through consolidation of redundant ligand entries.

This feedback, gathered through workshops and support requests, complements the usage and citation analyses and ensures that BRENDA evolves into a more user-oriented and accessible platform.

### Outlook

The analyses demonstrate that BRENDA is widely used and valued across all continents, while at the same time offering potential for further improvement (Fig. 4). Moreover, its usage is highly diverse, spanning different research areas such as scientific computing and biochemical research. Insights from user behavior, citation analyses, and direct community feedback will be directly incorporated into the redesign of the website and the modernization of the API. These measures will further strengthen BRENDA’s role as a central and sustainable resource in the life sciences.

## Data Availability

BRENDA (https://www.brenda-enzymes.org/) data can be freely downloaded in various formats (e.g. CSV, JSON, TXT) without restrictions, except that the origin of the data has to be properly cited when used in other works (CC BY 4.0 license). Registration is necessary to access data through the SOAP API, but registration is free of charge.
